# Supplementary figures and images for: Magnetically treated water for removal of surface contamination by Malathion on Chinese Kale (Brassica oleracea L.)
Source: PLoS One. 2024 May 17;19(5):e0298371. doi: 10.1371/journal.pone.0298371 (PMC11101036; doi:10.1371/journal.pone.0298371)

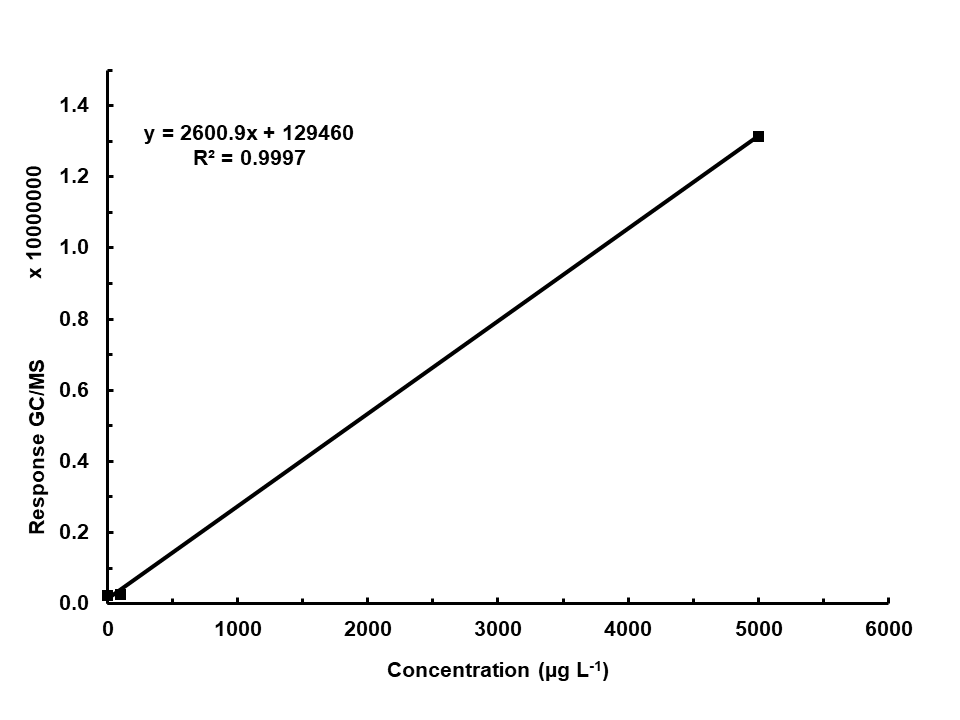

Supplement: S1 Fig — The calibration on authentic malathion is included in the S5 File. (TIF) [file pone.0298371.s001.TIF]

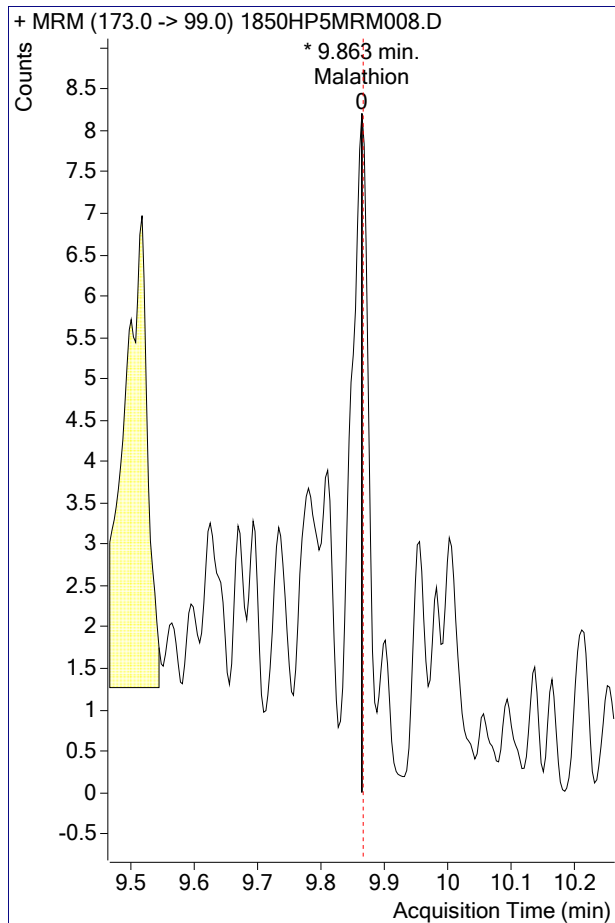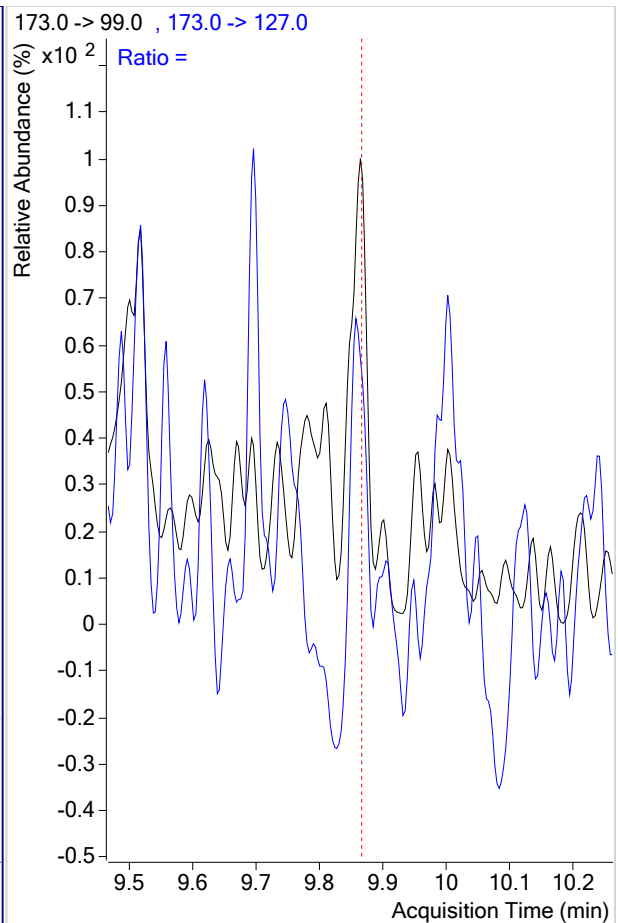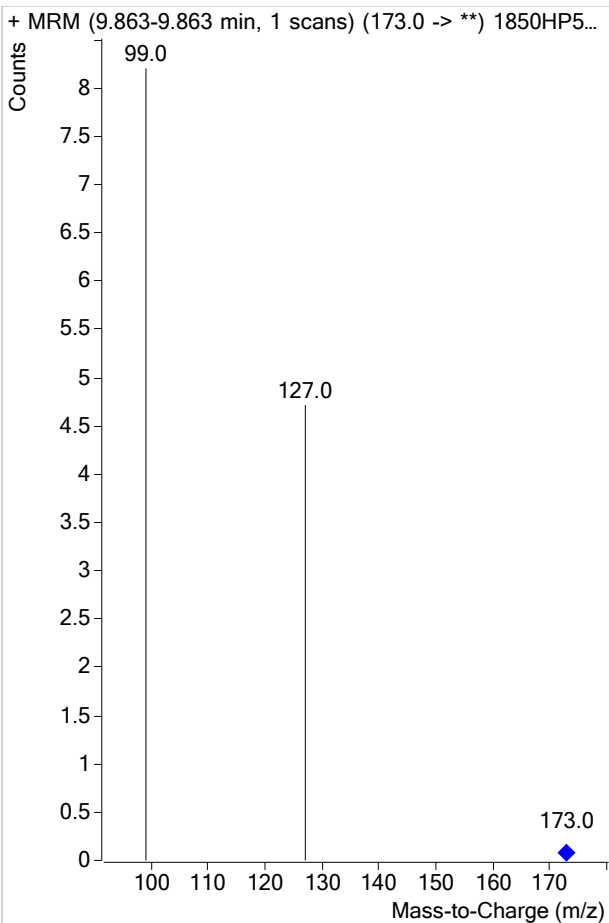

Supplement: S1 File — (PDF) [file pone.0298371.s002.pdf]

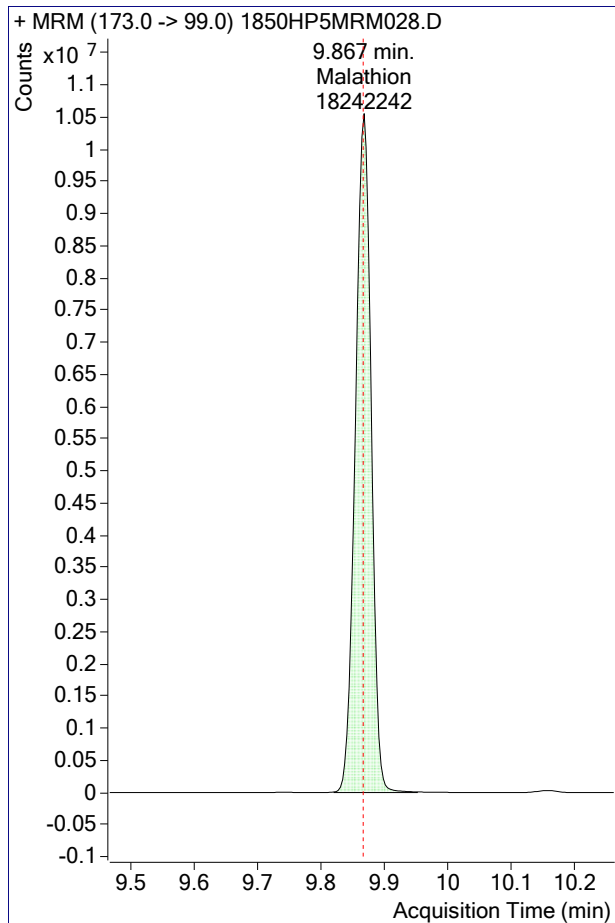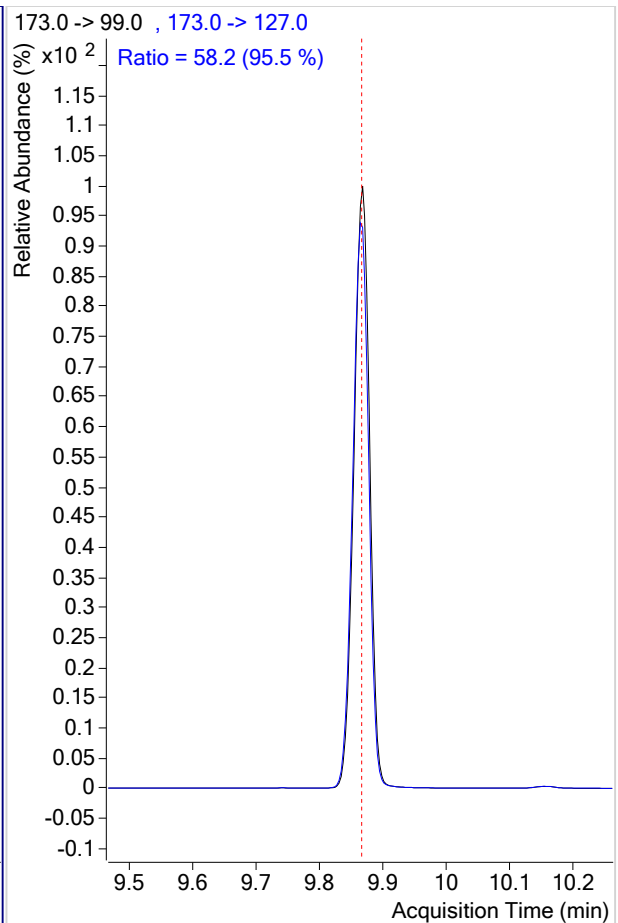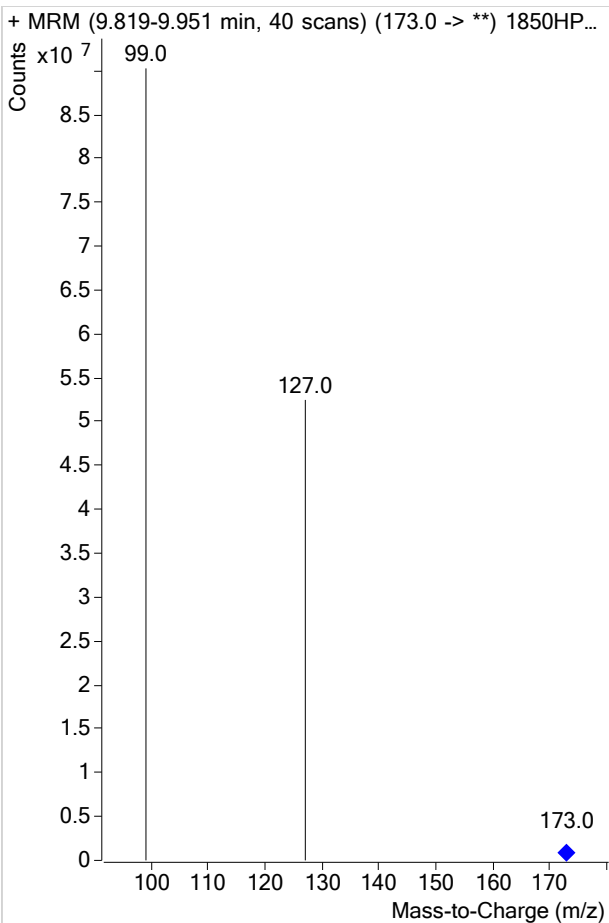

Supplement: S2 File — (PDF) [file pone.0298371.s003.pdf]

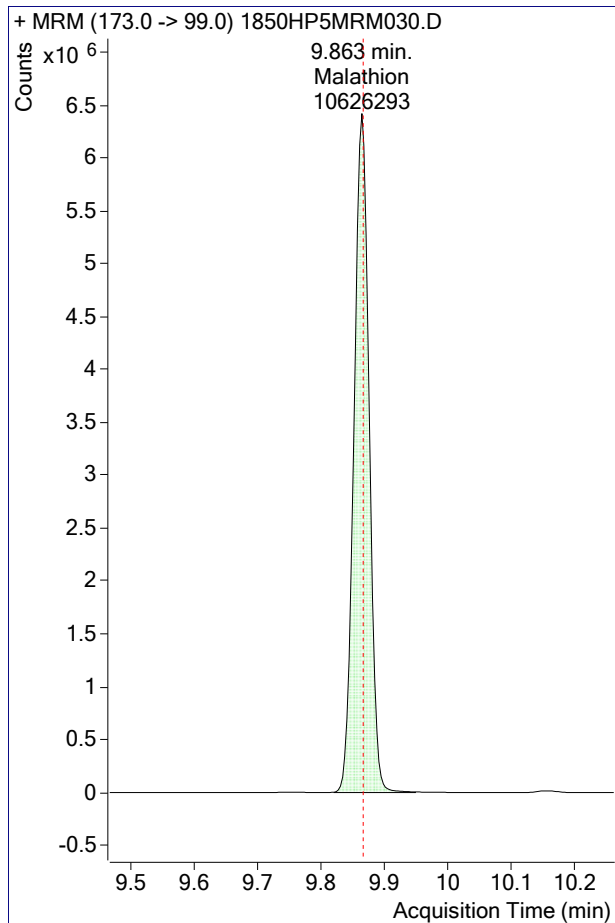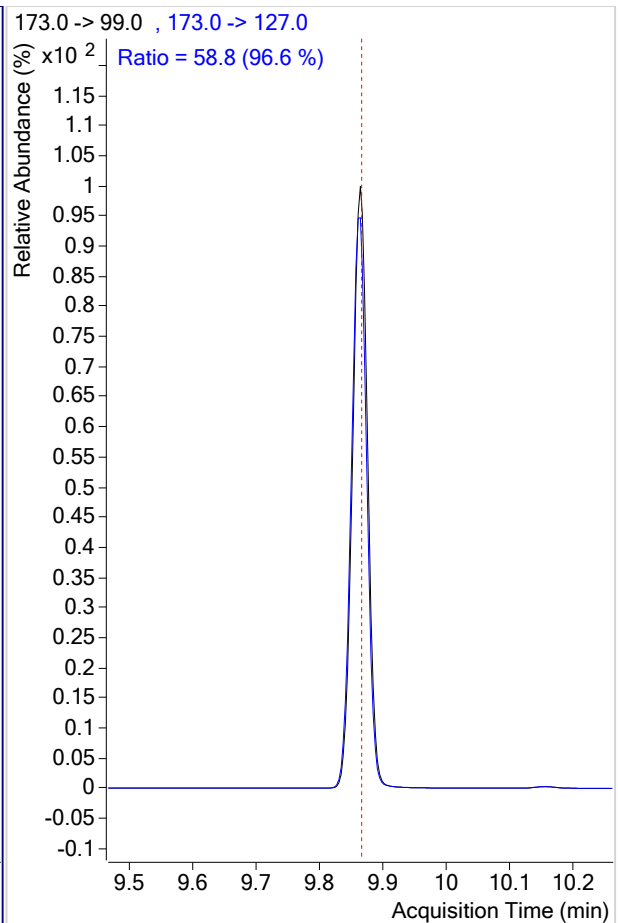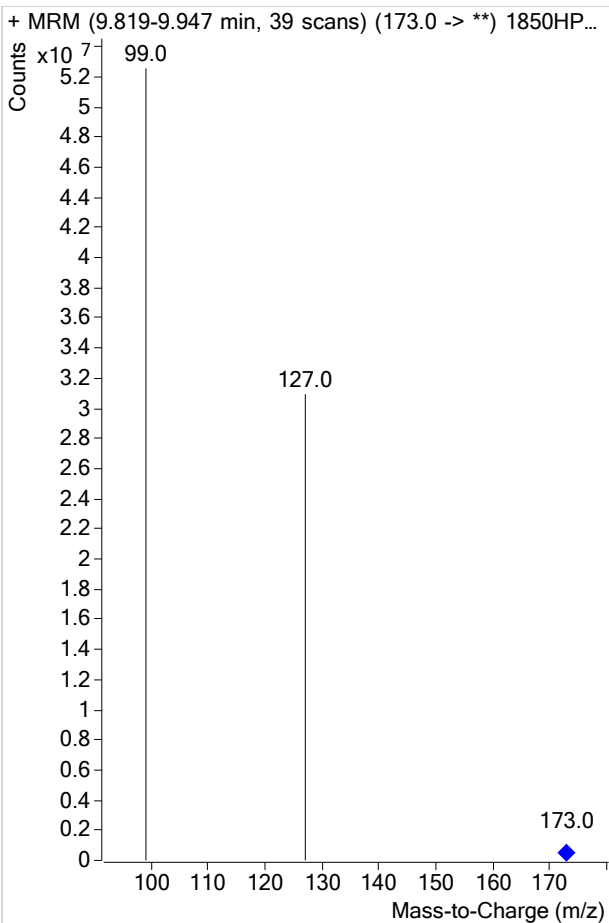

Supplement: S3 File — (PDF) [file pone.0298371.s004.pdf]

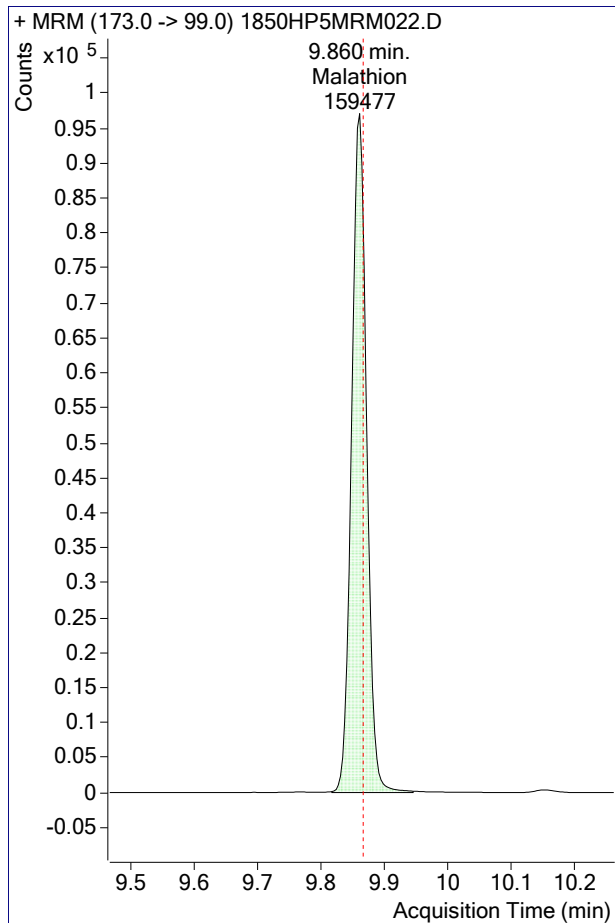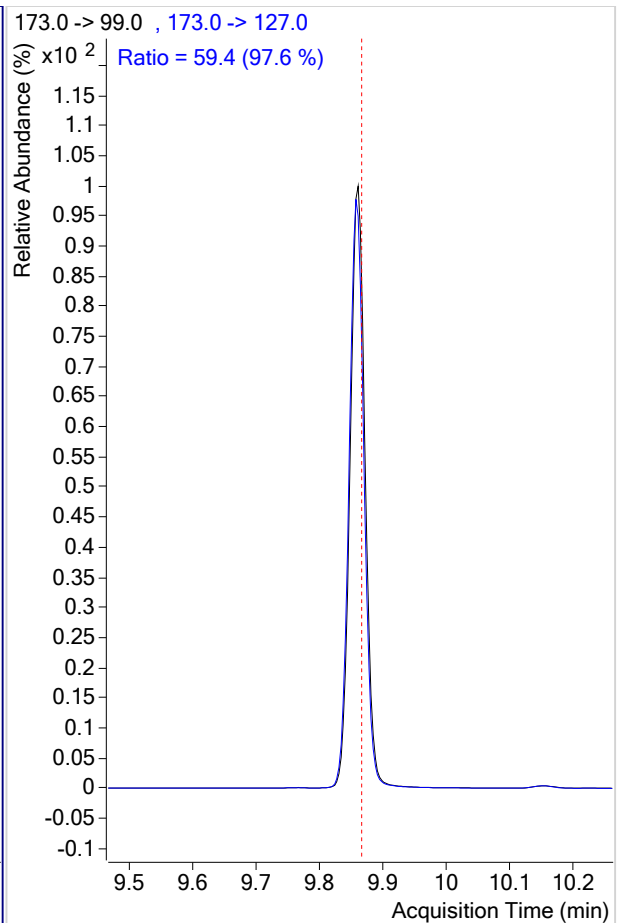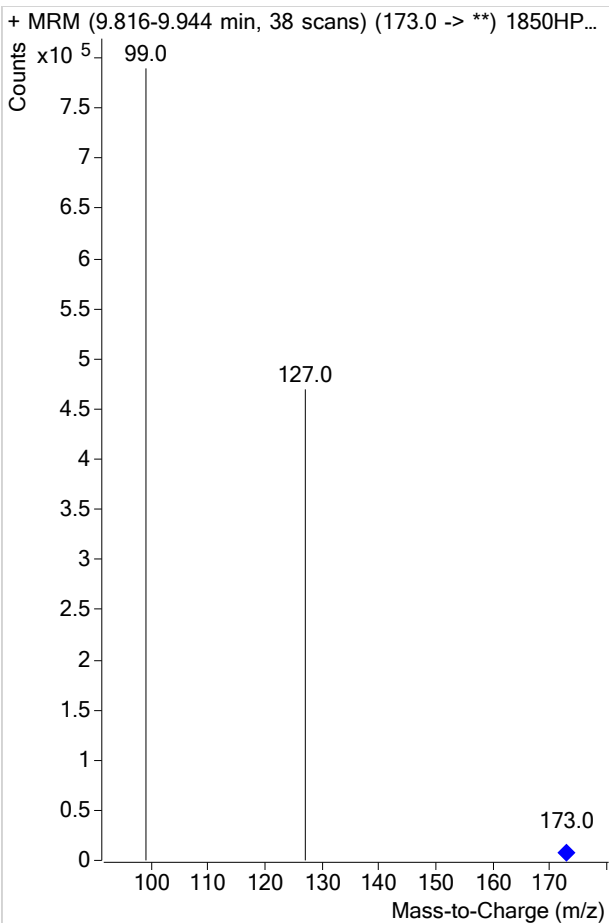

Supplement: S4 File — (PDF) [file pone.0298371.s005.pdf]
