# Supplementary material for: Magnetically treated water for removal of surface contamination by Malathion on Chinese Kale (Brassica oleracea L.)
Source: PLoS One. 2024 May 17;19(5):e0298371. doi: 10.1371/journal.pone.0298371 (PMC11101036; doi:10.1371/journal.pone.0298371)
Supplement: Attachment — Submitted filename: PONE - Final changes 12-dec-23.docx [file pone.0298371.s007.docx]

PONE-D-23-11448R1

Magnetically treated water for removal of surface contamination by Malathion® on Chinese Kale (Brassica oleracea L.)

Dr Raymond J. Ritchie

Dear Dr. Ritchie,

We've checked your submission and before we can proceed, we need you to address the following issues:

1. Please note that funding information should not appear in the Acknowledgments section/Funding section or Any other areas of your Manuscript. We will only publish funding information present in the Funding Statement section of the online submission form. Please

remove any funding-related text from the manuscript.

**L219-239 All funding information has been removed from the Acknowledgements etc.**

2. Please upload a Response to Reviewers letter which should include a point by point response to each of the points made by the Editor

and / or Reviewers. (This should be uploaded as a 'Response to Reviewers' file type.) Please follow this link for more information:

https://protect-au.mimecast.com/s/I7cHCvl1rKiED9oq6CQpjqF?domain=blogs.plos.org

**This has already been done. See previous change list.**

3. Please remove your figures/ from within your manuscript file, leaving only the individual TIFF/EPS image files. These will be automatically included in the reviewer’s PDF

**All figures in the clean version of the text have been removed as instructed.**

4. Your Data Availability statement currently reads:

"The attachments are the datafiles for figs 3 to 6."

Additionally, in your cover letter, you write:

"We have included the data sets as Supplementary material.

-Prompts (a) & (b) concerning data access. There is a difficulty with the Data Availability Statement. We the authors are happy to share our

data with colleagues however The Senior Author (Chadapust Sudsiri) informed me that the guidelines of the Research Development Grant

SIT6001026S does not allow us to deposit our data in a public repository."

And in the additional information section, you have written:

"All the data is available from the senior author (Chadapust J. SUDSIRI) upon request."

**To get around this problem we have included in the Supplementary Material full sets of the GC-MS data used for the project.**

Before we proceed, we’ll require some additional clarification to ensure your submission adheres to the PLOS ONE Data Availability policy

(https://protect-au.mimecast.com/s/7RH0CwV1vMfvDjXk1CqTXId?domain=journals.plos.org).

1) Please clarify whether your manuscript contains your minimal data set. The minimal data set is defined as the data set used to reach the conclusions drawn in the manuscript with related metadata and methods, and any additional data required to replicate the reported study findings in their entirety. This may include: a.) The values behind the means, standard deviations and other measures reported; b.) The

values used to build graphs; c.) The points extracted from images for analysis (https://protect-au.mimecast.com

/s/3fslCxngwOfL2lKDAcYJZSZ?domain=journals.plos.org).

**We confirm that the supplementary material contains all the necessary data.**

2) Please confirm whether there are legal or ethical restrictions on sharing your data publicly.

**Since we are providing all the necessary data as Supplementary Material we do not think there are any legal difficulties even though we are apparently not allowed to put the data in a repository.**

3) If legal or ethical restrictions apply, please provide all necessary instructions and non-author contact information (preferably email) for a data access committee, ethics committee, or other institutional body that other researchers would require to request access to your data.

Note that it is not acceptable for an author to be the sole named individual responsible for ensuring data access.

**We are providing data access by providing the data sets as Supplementary material**.

4) If there are no legal or ethical restrictions on sharing your data publicly, please upload the minimal anonymized data set necessary to replicate your study findings as either Supporting Information files, or to a stable public repository. If you upload your data to a repository,

please also provide the relevant URLs, DOIs, or accession numbers for other researchers to access your data directly. For a list of recommended public repositories, please see https://protect-au.mimecast.com

/s/JWlWCyojxQT7l1EKMCRwDnm?domain=journals.plos.org.

Once we receive this information, we will update your data availability statement on your behalf.

**Problem solved by inclusion of the data sets in the Supplementary material.**
